# Supplementary material for: From sequence to enzyme mechanism using multi-label machine learning
Source: BMC Bioinformatics. 2014 May 19;15:150. doi: 10.1186/1471-2105-15-150 (PMC4229970; doi:10.1186/1471-2105-15-150)
Supplement: Additional file 2 — Java code of ml2db. Additional file ml2db_code.tar.gz contains the Java source code to run the multi-label machine learning experiments and save the results to database. The code’s Javadoc is included. [file 1471-2105-15-150-S2.zip › additional file 2/ml2db/ml2db/doc/mulan/classifier/neural/model/class-use/ActivationFunction.html]

Uses of Class mulan.classifier.neural.model.ActivationFunction


JavaScript is disabled on your browser.


- Overview
- Package
- Class
- Use
- Tree
- Deprecated
- Index
- Help

- Prev
- Next

- Frames
- No Frames

- All Classes

## Uses of Class mulan.classifier.neural.model.ActivationFunction

- Packages that use ActivationFunction

  | Package | Description |
  | --- | --- |
  |  |  |
  | --- | --- |
  | mulan.classifier.neural.model |  |
- - ### Uses of ActivationFunction in mulan.classifier.neural.model

    Subclasses of ActivationFunction in mulan.classifier.neural.model

    | Modifier and Type | Class and Description |
    | --- | --- |
    |  |  |
    | --- | --- |
    | `class` | `ActivationLinear` Implements the linear activation function. |
    | `class` | `ActivationTANH` Implements the hyperbolic tangent activation function. |

    Methods in mulan.classifier.neural.model that return ActivationFunction

    | Modifier and Type | Method and Description |
    | --- | --- |
    |  |  |
    | --- | --- |
    | `ActivationFunction` | Neuron.`getActivationFunction()` Returns the `ActivationFunction` used by the `Neuron`. |

    Constructors in mulan.classifier.neural.model with parameters of type ActivationFunction

    Constructor and Description

    `Neuron(ActivationFunction function,
    int inputDim,
    double biasValue)`

    Creates a new `Neuron` instance.

    `Neuron(ActivationFunction function,
    int inputDim,
    double biasValue,
    java.util.Collection<Neuron> nextNeurons)`

    Creates a new `Neuron` instance.

    `Neuron(ActivationFunction function,
    int inputDim,
    double biasValue,
    java.util.Random random)`

    Creates a new `Neuron` instance.

    Constructor parameters in mulan.classifier.neural.model with type arguments of type ActivationFunction

    Constructor and Description

    `BasicNeuralNet(int[] netTopology,
    double biasInput,
    java.lang.Class<? extends ActivationFunction> activationFunction,
    java.util.Random random)`

    Creates a new `BasicNeuralNet` instance.

- Overview
- Package
- Class
- Use
- Tree
- Deprecated
- Index
- Help

- Prev
- Next

- Frames
- No Frames

- All Classes
